# Effect of Printing Parameters on Dimensional Error and Surface Roughness Obtained in Direct Ink Writing (DIW) Processes

**DOI:** 10.3390/ma13092157

**Published:** 2020-05-07

**Authors:** Irene Buj-Corral, Alejandro Domínguez-Fernández, Ana Gómez-Gejo

**Affiliations:** Department of Mechanical Engineering, Escola Tècnica Superior d’Enginyeria Industrial de Barcelona (ETSEIB), Universitat Politècnica de Catalunya (UPC), Av. Diagonal, 647, 08028 Barcelona, Spain; alejandro.dominguez-fernandez@upc.edu (A.D.-F.); agomezgejo@gmail.com (A.G.-G.)

**Keywords:** direct ink writing, surface roughness, dimensional error, infill, printing speed, layer height

## Abstract

Prostheses made from ceramic materials have the advantages of producing little debris and having good durability, compared with those made from metal and plastic. For example, hip prostheses require a porous external area that allows their fixation by means of osseointegration and a solid internal area that will be in contact with the femoral head. The manufacturing of complex ceramic shapes, by means of machining processes, for example, is complicated and can lead to breakage of the parts because of their fragility. The direct ink writing (DIW) process allows the printing of ceramic pastes into complex shapes that achieve their final strength after a heat treatment operation. This paper studies both the dimensional error and surface finish of porous zirconia prismatic parts prior to sintering. The variables considered are infill, layer height, printing speed, extrusion multiplier and bed temperature. The responses are the dimensional error of the lateral walls of the samples and an areal roughness parameter, the arithmetical mean height, Sa. Mathematical models are found for each response, and multiobjective optimization is carried out by means of the desirability function. The dimensional error depends mainly on the interaction between layer height and infill, while the roughness on the interaction between infill and printing speed. Thus, infill is an important factor for both responses. In the future, the behavior of compact printed parts will be addressed.

## 1. Introduction

Ceramic materials are widely used, with applications in, e.g., aerospace [1], electrical [2], chemical [3] and medical [4] fields. Prostheses are currently produced using metallic materials. However, they release debris and can sometimes cause local toxicity [5]. Another common material for prostheses is polyethylene; in many cases, metal and polyethylene are combined [6]. However, polyethylene shows a notable wear over time. Ceramics are not currently used extensively for prostheses for several reasons, including the difficulty involved in producing complex shapes by means of the machining process. Their brittleness and propensity to suffer from thermal shock are other disadvantages, as is their tendency to squeak. However, technical ceramics have specific properties that make them appropriate for this purpose [7]: they are hard, refractory and wear- and oxidation-resistant [8,9].

Direct ink writing (DIW) allows for the printing of ceramic parts from inks that are extruded, without the application of heat, then placed layer by layer on a printing bed [10]. For many ceramic materials, the process requires a subsequent sintering operation so that they can achieve their final strength. The sintering temperature is an especially important parameter that influences the mechanical strength of the parts. For example, in integrated ceramics it was observed that too low sintering temperatures led to low mechanical strength, while high sintering temperatures produced stresses and defects in the material [11]. The DIW process has certain advantages: it is easier to use and cheaper than other additive manufacturing processes, such as the photoprinting process, and has the potential for printing with a wide range of materials [12]. A further advantage is that since the viscosity of the DIW inks is often higher than that of melted plastic, fewer supports are required, except for high print-orientation angles [13]. A high level of surface roughness is attained in most extrusion processes, because of the stair-stepping effect [14]. In addition, quite high dimensional variability is observed. Some methods allow the material to be forced through a syringe in order to extrude it: pneumatic force, lever pressure force, etc. In this paper, gear wheels and a plunger are used. In extrusion processes, the use of different printing patterns and printing parameters leads to structures with required porosity and pore size values [15,16]. Finally, DIW is ideal for the production of compact parts [17,18].

In the past, several authors have used DIW to print ceramic parts (Table 1).

Few studies are available for DIW, so the influence of printing parameters on dimensional accuracy and roughness is largely unknown. Feilden et al. [17] used different tip diameters to print both SiC and Al_2_O_3_ ceramics and found that larger tips provided lower dimensional accuracy. The same authors noted that the surface finish is an important source of cracks that can lead to lower mechanical strength. Yu et al. [34] observed the shape of the stacked layers in the lateral walls of yttria-stabilized zirconia specimens, while Ra values higher than 30 μm were reported for yttria-stabilized zirconia with CeO_2_ with the syringe extrusion process [35].

However, several studies about dimensional accuracy and surface finish are found for a similar extrusion technique, in fused deposition modeling (FDM) or fused filament fabrication (FFF), mainly used for plastics. For example, Boschetto and Bottini [36] defined a design for manufacturing (DFM) methodology in order to improve the dimensional accuracy of the FDM processes. Rahman et al. [37] investigated the effect of bed temperature, nozzle temperature, printing speed, infill, layer thickness and the number of loops forming the shell of the parts on the dimensional deviations in X, Y and Z directions, as well as the arithmetical mean height of the profile Ra and vertical Ra. They printed acrylonitrile–butadiene–styrene (ABS) parts. They found that the optimum parameter settings for both dimensions and roughness corresponded to low bed temperature, low nozzle temperature, high printing speed, medium infill, low layer thickness and a low number of shells. Ceretti et al. varied the extrusion head type, the nominal size of pores and the displacement of the extrusion head on the Z-axis using polycaprolactone (PCL) [38]. They studied the dimensions of rectilinear grid structure scaffolds. The most influential parameter on the extruded diameter of the filament was the nominal size of the pores, while the resulting height of the pores was most influenced by the head type.

The ceramic prostheses usually require a dense area that will assure mechanical strength, combined with a porous area which will allow fixation of the implant by means of osseointegration [4]. The present paper addresses the performance of the porous structures and specifically aims to study and analyze the effect of different DIW printing parameters on porous ZrO_2_ specimens with respect to the surface roughness and dimensional error of their lateral walls. The five variables considered were the layer height, infill, printing speed, flow multiplier and printing bed temperature. Both responses were optimized by means of the desirability function. This study will help to minimize the dimensional error and surface roughness of the printed structures.

## 2. Materials and Methods

### 2.1. Printing Tests

**Figure 1:**
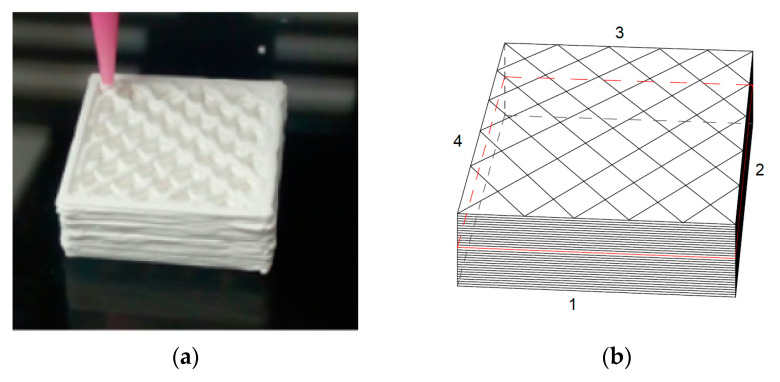
An example of a printed specimen: (**a**) picture; (**b**) schematic.

Prismatic samples of 20 × 20 × 10 mm^3^ were printed, with rectilinear infill pattern and raster angle of 45° (Figure 1a). The nozzle diameter was 0.67 mm. A dual-paste extruder from CIM-UPC was used.

The ink formulation was 40 vol % of ZrO_2_, with mean particle size of 40 μm. A pluronic acid solution of 25 wt % concentration was used.

Samples were heated to 100 °C for 5 h, in order to remove water.

### 2.2. Design of Experiments and Multiobjective Optimization

Design of experiments (DOE) consisted of a two-level five-variable fractional design (2^5−1^), with the following five variables: infill (IN), layer height (LH), printing speed (PS), extrusion multiplier (EM) and bed temperature (BT). Three central points were added to the design in order to assess the possible curvature of the models. The different experimental conditions are presented in Table 1. ANOVA was used to obtain regression models of the two responses under consideration.

The multiobjective optimization was carried out by means of the desirability function, which is a global equation that contains the desirability of the different responses [39]. Minitab17 software (Minitab, State College, PA, USA) was used for the statistical analysis.

### 2.3. Dimensional Error Measurement

The dimensions of the samples were measured with a Mitutoyo PJ300 profile projector (Kawasaki, Japan) to determine the dimensions of the four sides of the prismatic samples (numbered from 1 to 4 in Figure 1b).

For each side, the relative difference between the theoretical and the experimental dimension was calculated as a percentage [37]. The average value of the differences of the four sides was taken as the average dimensional error (%).

### 2.4. Roughness Measurement

Roughness was measured with Zeiss Smartproof 5 confocal equipment (Oberkochen, Germany). It uses white light to capture different two-dimensional images focused at different heights, and then combines them to obtain a surface topography. A 20× magnification lens was employed. Use of optical equipment avoids mechanical contact between the device and the samples, preventing them from being damaged. Optical methods have been used in the past in non-invasive evaluations of ceramic samples, for example to detect internal defects [11].

The areal arithmetical mean height parameter was considered (Equation (1)), according to the ISO25178 standard [40]. It corresponds to the average value of the differences in height, expressed as an absolute value, of each point compared with the central plane of the surface.

(1) $$\mathrm{Sa}\;=\frac{1}{A}\iint_{A}\left|Z\left(x,\;y\right)\right|\;\mathrm{dxdy}$$

where A is the measurement area and Z(x, y) is the function that defines the surface topography.

## 3. Results

### 3.1. Dimensional Error and Roughness

The average dimensional error and the areal arithmetic roughness, Sa, of the different experiments are shown in Table 2. The five variables considered are infill (IN), layer height (LH), printing speed (PS), extrusion multiplier (EM) and bed temperature (BT).

The highest dimensional error corresponds to experiments 3 and 7, printed with low infill, high layer height and low extrusion multiplier. The lowest dimensional error corresponds to experiments 8 and 16, printed with high infill, high layer height and high speed.

The lowest roughness values correspond to experiments 5, 7, 13 and 15, with low infill and high printing speed. This suggests that the interaction between infill and speed is very important. As a general trend, low infill with low speed provides high roughness values (experiments 1, 3, 9 and 11). A different combination of experiments with high infill and high speed also provides high roughness (experiments 6, 8, 14 and 16). Rahman et al. [37] reported similar results with medium infill (15%) and high speed (55 mm/s) in the FDM process of ABS parts.

**Figure 2:**
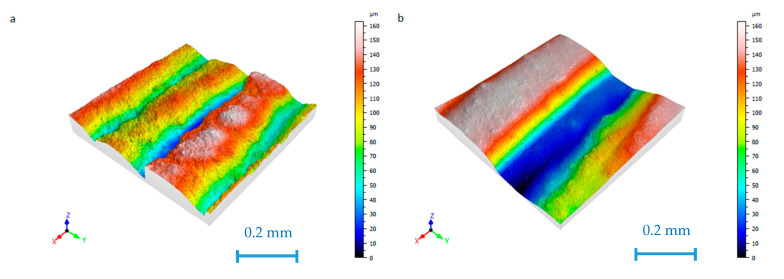
The surface topography of (**a**) sample 5 (Sa = 23.229 μm) and (**b**) sample 10 (Sa = 27.919 μm).

Different surface topographies were obtained on the lateral walls of the prismatic shapes. As an example, Figure 2 shows the surface topographies of samples 5 and 10.

Figure 2a, corresponding to sample 5, shows parallel crests which are consistent with the edges of the different printing layers, with round peaks and sharper valleys. In contrast, in Figure 2b, corresponding to sample 10, higher crests with wider and deeper valleys are observed, although the Sa value is only slightly higher than for sample 5.

### 3.2. Mathematical Model for Average Dimensional Error

A full linear model was obtained with adjusted R^2^ value of 91.90%. In order to simplify it, a reduced linear model was sought for average dimensional error, which is presented in Equation (2), with an adjusted R^2^ value of 69.71%.

Dim. Error = −17.90 + 0.0849 IN + 47.0 LH + 12.03 EM + 0.0466 BT − 0.2004 IN·LH − 0.000869 IN·BT − 31.4 LH·EM(2)

**Figure 3:**
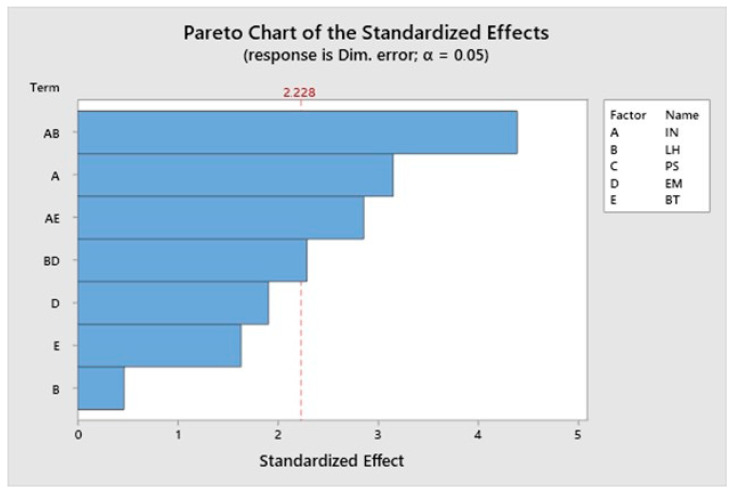
The Pareto chart of the standardized effects for average dimensional error.

The Pareto chart for average dimensional error is presented in Figure 3.

**Figure 4:**
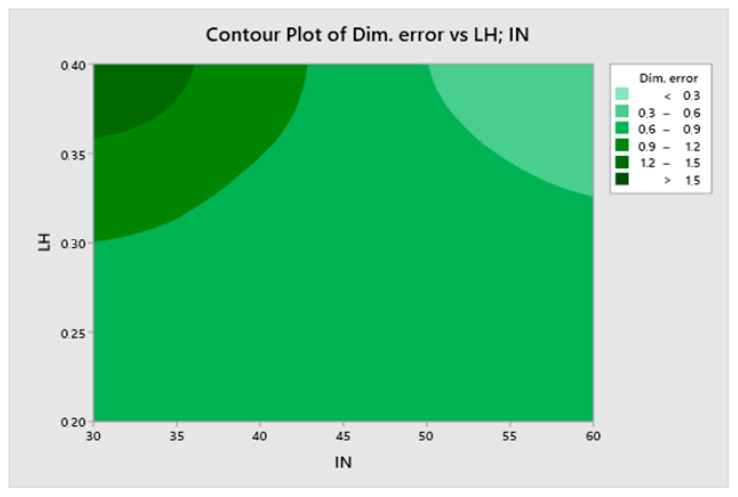
A contour plot of dimensional error (%) vs. infill and layer height.

The most significant effect on the dimensional error is the interaction between infill and layer height, followed by infill and then by the interaction between infill and temperature. Printing speed is not a significant factor for the dimensional error. Figure 4 corresponds to a contour plot of the dimensional error vs. infill and layer height.

The lowest dimensional error corresponds to high infill with high layer height. However, a high dimensional error is obtained with low infill and high layer height. Low layer height provides quite a low dimensional error regardless of infill.

### 3.3. Mathematical Model for Sa

A full linear model was obtained for roughness parameter Sa, with adjusted R^2^ value of 81.56%. A reduced linear model for areal arithmetic roughness Sa is presented in Equation (3), with an adjusted R^2^ value of 63.91%.

Sa = 48.7 − 0.538 IN + 89.6 LH − 8.89 PS + 0.245 BT + 0.1224 IN·PS − 1.856 LH·BT + 0.0563 PS·BT(3)

**Figure 5:**
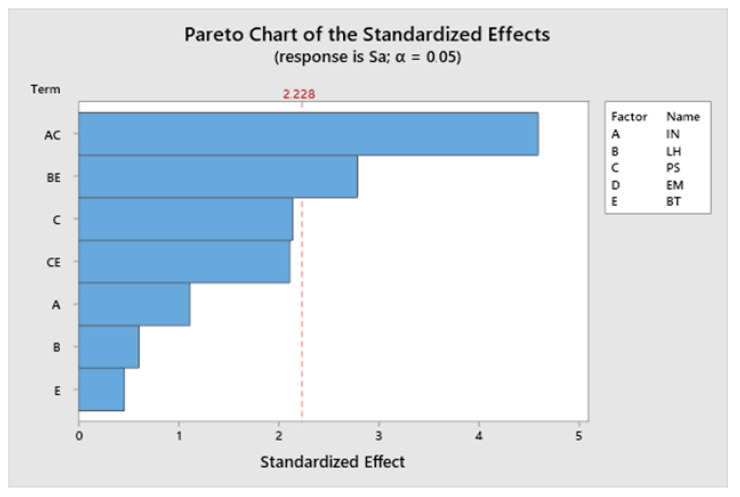
The Pareto chart of standardized effects for Sa.

The Pareto chart for Sa is shown in Figure 5, for α = 0.05.

**Figure 6:**
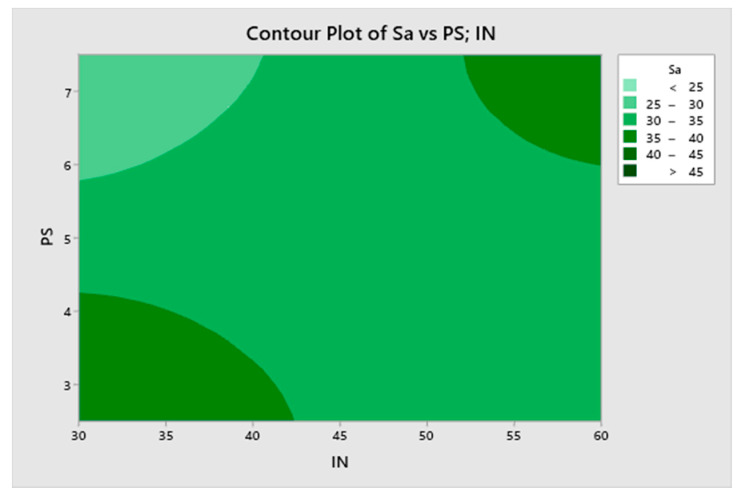
A contour plot of mean arithmetical roughness Sa (μm) vs. infill and printing speed.

The most significant term is the interaction between infill and printing speed, followed by the interaction between layer height and temperature. The extrusion multiplier has no significant effect on roughness. Figure 6 depicts a contour plot of Sa vs. infill and printing speed, whose interaction is the most significant term of the model.

The lowest roughness values are obtained with high printing speed and low infill. In contrast, both combinations of low infill/low speed and high infill/high speed lead to higher roughness values.

### 3.4. Multiobjective Optimization

The results of the multiobjective optimization of roughness and dimensional error are presented in Table 3. First, the same importance is given to each of the two responses: average dimensional error and Sa. Then, one of the responses is given a higher importance than the other.

Similar results were obtained if the same importance is considered for both responses or if roughness is given higher importance: low infill, low layer height, high printing speed, and low temperature. However, if dimensional error is given higher importance, the results are opposite, with high infill, high layer height, low printing speed and high temperature.

## 4. Discussion

One of the main advantages of printed prostheses is the potential for manufacturing customized parts. Dimensions have a major influence on the loosening of prostheses. For example, in acetabular cups, use of a small diameter acetabular cup with cemented fixation has been identified as a potential loosening risk, because in small parts the mechanical stresses increase at the bone–cement interface [41]. Thus, dimensions of the prostheses have an important effect on their survival rate, and the measurement of dimensional error is recommended.

Surface roughness influences the performance of the ceramic prostheses [16]. For example, the internal walls of hip prostheses require a smooth surface because of the connection with the femoral head. In order to achieve such a smooth surface, a polishing operation is usually required [42]. Since, material is deposited layer by layer in the DIW printing process, the vertical and inclined walls of the specimens will show higher roughness than the horizontal ones. This effect has been studied both for FDM-printed plastic materials [15] and for ceramic materials [34]. If low roughness could be achieved by the printing process, the need for a subsequent polishing operation would be reduced or even eliminated. For this reason, it is important to determine the surface finish values obtained in lateral walls of DIW-printed parts.

Other authors have studied dimensional error and/or surface roughness of extrusion-printed parts, mainly by the FDM process. Messimer et al. [43] reported dimensional errors of up to 3% for high-temperature polylactic acid (HTPLA), which are higher than those obtained in this study. Rahman et al. [37] reported similar dimensional error values, and Ra values in vertical walls of up to 28 μm for ABS parts, for layer height between 0.2 and 0.4 mm. Ra values of around 20 µm were obtained in lateral walls of cylindrical shapes, for a layer height of 0.25 mm [15]. These values are on the same order of magnitude as those obtained in the present work for ceramics. Although DIW has been used in the past to print ceramics [17,20,44,45], few studies have addressed both the roughness and the dimensional error of DIW-printed ceramic samples. The roughness values obtained in the present work are similar to those reported by de Luis [35], with Ra = 30 μm, for ytrria-stabilized zirconia with CeO_2_ in syringe extrusion processes.

In the present work, the most influential terms on dimensional error are infill and the interactions between infill and layer height and between infill and temperature. According to García Plaza et al., neither feed rate nor layer thickness influenced dimensional error significantly in FDM-printed plastic parts [46]. High infill combined with high layer height resulted in the lowest dimensional error. This might be attributed to the fact that, in this case, more compact samples were obtained with lower dimensional variability.

As far as roughness is concerned, unlike the FDM process, where layers have a more regular structure [15] and layer height is usually an important influencing factor [47], more irregular surfaces are obtained in the present work (Figure 3) and some interactions between variables are observed. Thus, the interaction between infill and printing speed and the interaction between layer height and temperature are the most influential parameters on roughness. Specifically, low infill combined with high printing speed leads to lowest roughness values. Low infill implies less material to be deposited on each layer, while high printing speed reduces printing time, thus preventing ink-drying and promoting the correct deposition of the ceramic layers. 

In this paper, surface finish and the dimensional error of porous ceramic structures were studied. In future studies, compact structures will be manufactured, with low porosity, in order to assess the mechanical strength of the DIW-printed parts. The presence of cracks or pores reduces the mechanical strength of ceramic samples [47].

## 5. Conclusions

In this paper, porous zirconia prismatic samples were printed by means of direct ink writing (DIW). Both the dimensional error and roughness were studied and analyzed. The main conclusions of the paper are summarized below:

- The average dimensional error of the samples ranged from 0.14% to 1.49%. The results are comparable to those stated in the literature for FDM technology. High infill and high layer height are recommended in order to decrease the dimensional error.
- Areal average roughness Sa ranged from 25 to 43 µm. The results obtained were similar to those found by other authors for both DIW and FDM technologies. Low infill and high printing speed are recommended in order to reduce surface roughness.
- According to multiobjective optimization, low infill, low layer height and high printing speed are recommended if both responses are given the same importance, or if roughness is more important than dimensional error. However, if dimensional error is more important than roughness, then high infill, high layer height and low printing speed are recommended.

The results presented in this paper will assist in the selection of the appropriate printing parameters in DIW processes for ceramics, when dimensional error and roughness are to be minimized. This will help to manufacture customized ceramic parts such as prostheses.

## Figures and Tables

**Table 1:** A summary of recent research on direct ink writing (DIW)-printed ceramic parts.

Application	Detailed Ceramic Composition	Authors	Year of Publication	References
Manufacture of interpenetration phase composites	Al_2_O_3_ and ZrO_2_, with Al infiltration	San Marchi et al.	2003	[19]
Orthopedic applications	Β-Tricalcium phosphate	Miranda et al.	2006	[20]
Semiconductors	BaTiO_3_	Sun et al.	2009	[21]
Bone repair	Polycaprolactone/hydroxyapatite	Xu et al.	2014	[22]
Biomedical engineering	SiC/Al_2_O_3_	Feilden et al.	2016	[17]
Different engineering applications	Yttria-stabilized zirconia	Peng et al.	2017	[23]
Thermoelectric materials	Conductive acrylonitrile butadiene styrene (CABS)-ZnO	Aw et al.	2018	[24]
Traditional ceramic industry	Kaolinite clay	Revelo and Colorado	2018	[25]
Prostheses	Zirconia toughened alumina	Stanciuc et al.	2018	[26]
Structural applications	Yttria-stabilized tetragonal zirconia polycrystal	Li et al.	2018	[27]
Laser lenses	YAG/Nd:YAG	Jones et al.	2018	[28]
Electronic packaging field	Plated copper ceramic substrates with kaolin suspensions	Sun et al.	2019	[29]
Filters, catalyst supports, thermal insulators	Si_2_N_2_O	Jin et al.	2019	[30]
Structural and heat resistant materials	Carbon fiber reinforced SiC	Lu et al.	2019	[31]
Bone designs	Hydroxyapatite	Roopavath et al.	2019	[32]
Bone tissue engineering	Hardystonite scaffolds	Elsayed et al.	2019	[33]

**Table 2:** The experiments and the results for roughness and dimensional error.

No.	IN (%)	LH (mm)	PS (mm/s)	EM	BT (°C)	Dimensional Error (%)	Sa (μm)
1	30	0.2	2.5	1.15	60	0.74	38.486
2	50	0.2	2.5	1.15	30	0.48	27.435
3	30	0.4	2.5	1.15	30	1.47	42.937
4	50	0.4	2.5	1.15	60	0.26	29.249
5	30	0.2	7.5	1.15	30	0.50	23.229
6	50	0.2	7.5	1.15	60	0.34	46.999
7	30	0.4	7.5	1.15	60	1.49	25.937
8	50	0.4	7.5	1.15	30	0.14	36.416
9	30	0.2	2.5	1.25	30	0.29	33.411
10	50	0.2	2.5	1.25	60	1.14	27.919
11	30	0.4	2.5	1.25	60	1.66	34.751
12	50	0.4	2.5	1.25	30	0.60	42.188
13	30	0.2	7.5	1.25	60	1.34	25.605
14	50	0.2	7.5	1.25	30	1.39	31.321
15	30	0.4	7.5	1.25	30	0.70	25.987
16	50	0.4	7.5	1.25	60	0.20	31.628
17	40	0.3	5.0	1.20	45	0.84	33.034
18	40	0.3	5.0	1.20	45	0.70	31.449
19	40	0.3	5.0	1.20	45	0.95	36.992

**Table 3:** The experiments and results for roughness and dimensional error.

Importance of Sa:Importance of Dimensional Error	IN (%)	LH (mm)	PS (mm/s)	BT (°C)	Composite Desirability
1:1	30	0.2	7.5	30	0.954
10:1	30	0.2	7.5	30	0.983
1:10	50	0.4	2.5	60	0.962
